# Pucker sign in proximal humerus fracture: Case report and review of the literature

**DOI:** 10.1016/j.tcr.2026.101402

**Published:** 2026-05-21

**Authors:** Lorenzo Lucchetta, David Palmieri, Michele Bisaccia, Giuseppe Rinonapoli

**Affiliations:** a Orthopedics and Traumatology Department, University of Perugia, S. Maria della Misericordia Hospital, Piazzale Menghini, 06129, Perugia, Italy

**Keywords:** Pucker sign, Proximal humerus fracture, Traumathology

## Abstract

**Introduction:**

Proximal humerus fractures (PHFs) are among the most common upper limb fractures, particularly in elderly patients after low-energy falls. The *pucker sign* is a rare but clinically important finding caused by penetration of a fracture fragment through the deltoid muscle, leading to entrapment of subcutaneous tissue and visible skin dimpling. This sign indicates soft tissue interposition that may prevent closed reduction and threaten skin vascularity if unrecognized.

**Case report:**

A 31-year-old male with a history of multiple sclerosis presented with a proximal humerus fracture (AO/OTA 11A2.2) and an evident pucker sign following a low-energy fall. Closed reduction under mild sedation was successfully achieved, followed by intramedullary nailing without complications. Postoperative recovery was uneventful, and early mobilization was initiated, although long-term follow-up was unavailable due to patient relocation.

**Discussion:**

A systematic literature review identified twelve published cases of PHFs associated with a pucker sign. The mean patient age was 46.3 years, with equal sex distribution. Closed reduction succeeded in 41.7% of cases, whereas 58.3% required open reduction and internal fixation. Successful closed reduction was generally limited to minimally displaced extra-articular fractures (AO/OTA 11A2–A3), while fully displaced fractures consistently required open management. Early recognition and sedation-assisted reduction improve outcomes and prevent skin necrosis or neurovascular compromise.

**Conclusion:**

The pucker sign in proximal humerus fractures is a rare but significant indicator of soft tissue entrapment. Prompt identification and timely reduction, closed or open, are essential to avoid complications. Increased awareness and additional case reporting are needed to establish standardized management strategies for this uncommon but clinically relevant presentation.

## Introduction

Proximal humerus fractures (PHFs) are the most common fractures of the humerus and account for about 6 fractures per 10′000 patients [1], [2], [3]. Their incidence rises with age, occurring three times more often in women than in men. Most PHFs result from low-energy falls, particularly among elderly patients. While most fractures are closed and un-displaced, around one-fifth require surgery [4].

The pucker sign results from a unique mechanism where a fracture fragment penetrates the deltoid muscle and becomes lodged in the subdermal layer, creating a visible dimple. [5]

Alshryda et al. [6] described this phenomenon as the distal fragment “buttonholing” through the deltoid [6] The resulting skin puckering indicates interposition of soft tissues that prevents closed reduction [4]. Hughes et al. suggested that even low-energy injuries might create enough shearing force to allow the metaphyseal spike to slide between deltoid fibers [7]. The skin over the incarcerated fragment indicates possible soft-tissue incarceration and skin vascularization compromise; delayed management can lead to necrosis or progression to an open fracture [8]. Because muscle tension tends to draw the skin more tightly around the fragment, attempts at closed manipulation often may worsen the puckering [5].

The purpose of this article is to report a new case of pucker sign in a PHFs and to review the literature to summarizing the clinical presentation, diagnosis and management of the cases in which the pucker sign is found.

## Case report

**Fig. 1:**
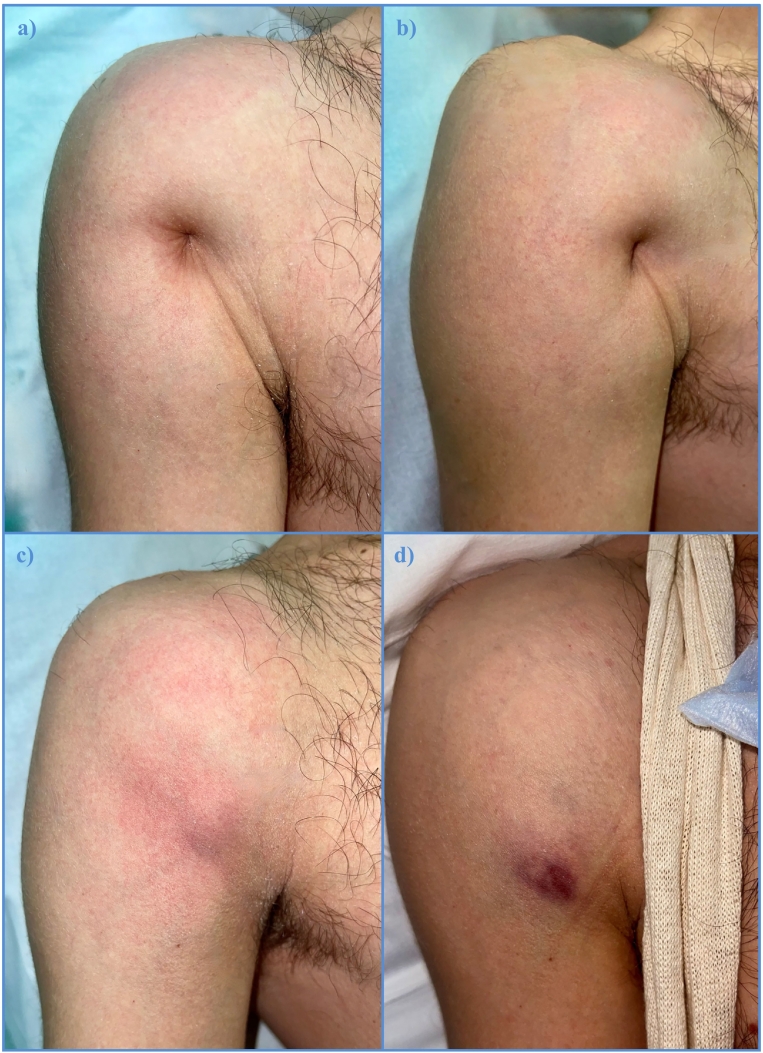
a-b) Pucker sign c) immediately after closed reduction d) 6 hours after reduction in Gilchrist bandage.

**Fig. 2:**
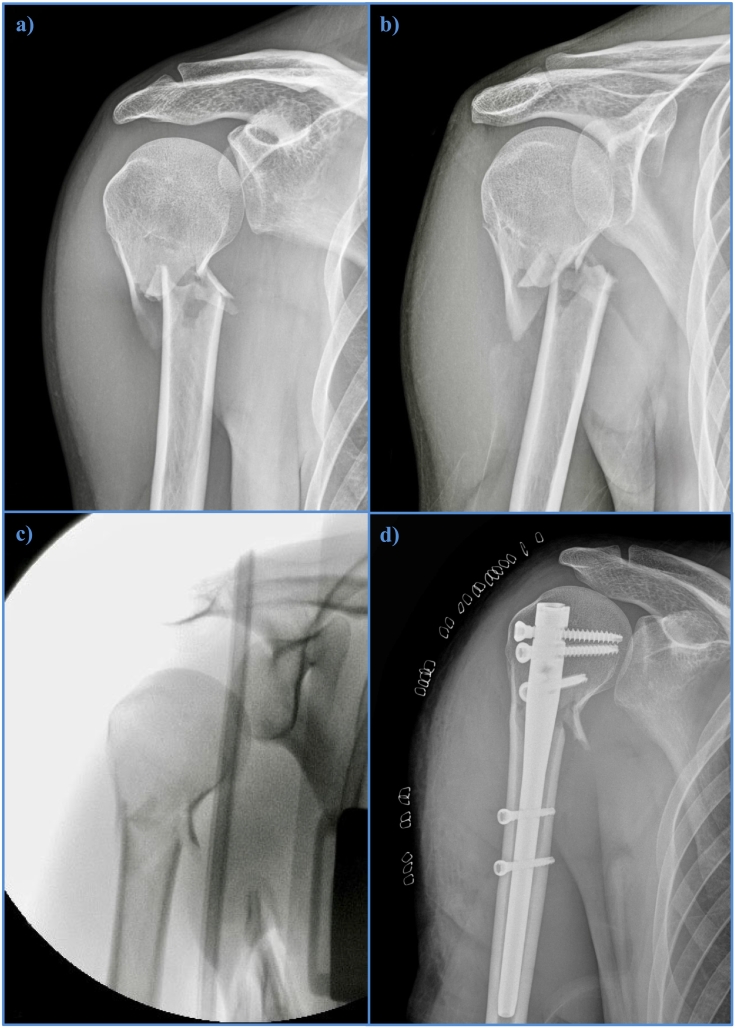
a-b) Fracture pattern c) after closed reduction d) post-operative X-rays.

A 31-year-old male with a known history of multiple sclerosis presented to the emergency department following a simple fall on the right shoulder after slipping from the stairs. The patient was triaged and then referred to the radiologist department to obtain plain Radiographs and then immediately after to the orthopedic emergency service for further evaluation. Clinical examination revealed a positive Pucker sign over the right shoulder, (Fig. 1) corresponding to a radiographically proximal humerus fracture (type 11A2.2 according to the AO/OTA classification). There were no open wounds or external lesions, distal pulses were palpable, and sensitivity was preserved. After obtaining informed consent, the patient was sedated under the supervision of an anesthetist with ventilatory support, and a closed reduction of the fracture was successfully performed under mild sedation. Following reduction, a Gilchrist bandage was applied and maintained until the following day. On the next day, the patient underwent surgical fixation with an intramedullary nail. Intra and post-operative images are showed in Fig. 2.

During the surgery, the reduction was achieved without major technical difficulties, as the fracture was previously reduced, and the procedure did not require the opening of a surgical window to remove interposed bone fragments between the fracture. The surgery was completed without intraoperative complications.

Postoperatively, the patient was instructed to immobilize the shoulder with a brace for 14 days but with the instruction to remove the brace 2–3 times per day to passively move the shoulder and the elbow to prevent post-operative stiffness. Clinical recovery was satisfactory; however, the patient was lost to follow-up due to residing in another region.

## Review of reported cases in PHFs

A comprehensive literature search was conducted across the main medical databases, including PubMed, Scopus, and Google Scholar, using combinations of keywords such as “*proximal humerus fracture*,” “*pucker sign*,” “*skin dimpling*,” and “*soft tissue interposition*.” Reference lists of relevant articles were also screened to identify additional reports. After applying reviewing the literature nine articles on proximal humerus fractures (PHFs) presenting with a pucker sign were identified across the retrieved literature.

The overall mean age at presentation was 46.3 years (range 16–78 years) and the patients were six women and six men. According to the AO/OTA system, three cases were classified as type 11A3 (including one subtype 11A3.2), four as type 11A2.2 (one associated with a Gustilo–Anderson I open fracture), two as type 11A2.1 and one as type 11A2.3; in two cases the classification was not reported and the radiographs were not accessible by the authors.

Closed reduction succeeded in five of the twelve patients (41.7%), whereas in seven cases (58.3%) authors proceeded directly to surgical management without an initial closed reduction attempt. Eight patients (66.7%) required open reduction and internal fixation: plating was used in five cases, Kirschner wires in two and an intramedullary nail in the newly added patients of this case report. The other four cases were managed non-operatively or with closed reduction alone.

Table 1 summarizes the reported cases of pucker sings' in PHFs.

**Table 1:** Reported cases of Pucker Sings's PHFs.

Reference	Age/Sex	Comorbidities	Trauma	AO	Treatment	Outcome	Notes
Alshryda et al. 2008 [6]	53/W	/	Fell from a ladder directly on shoulder	11A3	No closed reduction - ORIF Plate	Full recovery	First Report. Distal fragment through deltoid
Robinson et al. 2011 [12]	63/F	/	Simple fall	/	No Closed Reduction ORIF -Plate	Full recovery	/
	53/M	Angina	Simple fall	/	Closed reduction. ORIF -Plate	Peri-plate fracture	/
Davarinos et al. 2011[11]	19/W	Smoker, 4 weeks post-partum	Simple fall	11A2.2	No closed reduction ORIF- Plate	Full recovery	Distal fragment through deltoid
Aneja et al. 2012 [9]	46/W	/	Simple fall	11A2.1	Closed reduction	Lost to Follow-up	/
Jindal et al. 2013 [5]	16/M	/	Simple fall	11A2.1	No closed reduction. ORIF- Kirschner wires	Full recovery	Distal fragment through deltoid
	17/M	/	Traffic accident	11A3.2	No closed reduction. ORIF- Kirschner wires	Full recovery	Distal fragment through deltoid
Hughes et al. 2016 [7]	58/M	Heavy smoker and alcohol abuser	Fall from aground floor window	11A3	Closed reduction after 12 h – Conservative	Lost to Follow-up	/
Mellick et al. 2016 [10]	43/W	Cocaine Abuser	Fall from a mini barstool	11A2.3	Closed reduction	Lost to Follow-up	/
Olive et al. 2021 [8]	78/M	Pacemaker due to bifascicular block.	Simple fall	11A2.2	Conservative treatment	Full recovery	Unique case of spontaneous un-puckering after 24 h
Huyke-Hernandez et al. 2022 [15]	78/F	Past cerebrovascular accident, IAS, chronic kidney disease	Simple fall	11A2.2 Gustilo-Anderson I	Early antibiotics and 36 h after ORIF -Plate. Pucker debridement	Full recovery	Distal fragment through deltoid and skin
Lucchetta et al. 2025	31/M	Multiple Sclerosis	Simple fall	11A2.2	Closed reduction. ORIF-Nail	Full recovery	/

## Clinical presentations

Patients with pucker sign usually present with severe pain, swelling and a noticeable dimple over the anterior shoulder. The skin overlying the fracture appears puckered or tented, often with ecchymosis. Neurovascular examination should assess sensation over the deltoid (axillary nerve) and distal pulses, as incarceration could compromise these structures [8]. Radiographs typically show a fracture of the surgical neck of the humerus, sometimes with minimal displacement [7]. Imaging does not reveal skin entrapment, but persistent puckering after gentle traction indicates soft-tissue incarceration. Jindal et al. proposed ultrasound to detect interposed tissues [5].

## Management

The strategies depend on the extent of fracture displacement, skin viability and neurovascular compromise. In most reported cases, the pucker sign mandates open reduction and internal fixation. Alshryda et al. treated three patients with Plate ORIF after unsuccessful closed reduction [6]. Jindal et al. emphasised that the pucker sign denotes a “fracture of necessity” requiring open surgery [5]. Intra-operatively, the incarcerated skin must be carefully freed, and the fracture reduced before fixation.

Closed reduction may succeed when the fracture is minimally displaced and the skin remains viable. Hughes et al. reported a case where gentle axial traction relieved the puckering and allowed ORIF [7]. Olive et al. described an exceptional case of spontaneous un-puckering, where the dimple resolved after 24 h of immobilisation and analgesia, permitting conservative treatment [8]. However, spontaneous resolution is rare, and observation beyond 12–24 h risks skin necrosis [8]. A reasonable algorithm is: initial assessment and immobilisation; closed management may be attempted but under patient sedation. If puckering persists early open reduction with or without ORIF is indicated. Prophylactic antibiotics are recommended when skin viability is uncertain.

## Discussion

The pucker sign in humerus fractures remains a rare but significant clinical condition. Recognizing this sign is crucial because it signals soft-tissue interposition between bone fragments and potential neurovascular compromise. The mechanism involves buttonholing of the fracture fragment through the deltoid, leading to incarceration of skin and fascia [6]. While proximal humerus fractures are common among elderly women following low-energy falls [1], the pucker sign appears primarily in displaced surgical neck fractures, often in younger adults [7].

The limited number of reports suggests that this sign is exceedingly rare, but it may be under-recognised.

In the present case series, all instances of successful or attempted closed reduction occurred in minimally displaced extra-articular fractures according to the AO/OTA 11A2–A3 classification, corresponding to two-part surgical neck fractures in the Neer system. Specifically, the cases by Aneja et al. [9] [11A2.1], Hughes et al. [7] [11A3], Mellick et al. [10][11A2.3], and this case report [11A2.2] demonstrated that closed reduction could be achieved, either temporarily or definitively, when the distal fragment remained impacted beneath the deltoid without complete displacement or rotation.

These patterns suggest that the potential for closed reduction in pucker sign fractures is limited to extra-articular metaphyseal fractures (A2–A3) with preserved cortical contact and minimal soft-tissue interposition. Conversely, fully displaced fractures consistently required open reduction due to the impalement of the distal fragment through the deltoid and subcutaneous tissues. [5], [6], [11], [12]

It is essential that any attempt at closed reduction be performed under mild sedation and anesthetic supervision, as muscle relaxation significantly improves the likelihood of success by reducing deltoid tension and allowing safer disengagement of the trapped fragment. [13] Inadequate relaxation or excessive traction, on the other hand, may aggravate soft-tissue incarceration or cause secondary displacement, ultimately necessitating open reduction [5].

In only one reported case by Olive et al. [8], spontaneous un-puckering can occur but should not delay intervention longer than 24 h. Beside this, the management of pucker sign generally necessitates early closed reduction or if failed, open reduction and fixation.

Prophylactic antibiotics and early reduction are advisable when skin viability is uncertain [14]. Once the fragment is reduced and fixation achieved, prognosis is generally good; no report has documented long-term functional deficits attributable to the pucker sign itself.

The rarity of this sign precludes prospective studies; thus, management recommendations are based on case reports and small case series. Nonetheless, the consistent theme across publications is that the pucker sign is an alarm for soft-tissue interposition and failure of closed reduction. It should be distinguished from superficial skin wrinkling due to swelling or contusion. Further reporting of cases and development of guidelines could improve understanding and treatment of this uncommon presentation.

## Conclusion

The pucker sign associated with humeral fractures is a rare clinical sign indicating that a fracture fragment has penetrated soft tissues and trapped the skin. While the majority of proximal humerus fractures can be managed conservatively [1], the presence of a pucker sign generally indicates the necessity of open reduction and internal fixation to prevent cutaneous necrosis and neurovascular injury. Early recognition and timely surgical intervention are key to successful outcomes. Although occasional cases of spontaneous un-puckering or successful closed reduction have been described [7], [8], these remain exceptions rather than the rule. Clinicians should maintain a high index of suspicion for this sign and manage patients accordingly.

## Abbreviations

AO/OTA: Arbeitsgemeinschaft für Osteosynthesefragen/Orthopedic Trauma Association classification system
ED: Emergency Department
MS: Multiple Sclerosis
ORIF: Open Reduction and Internal Fixation
PHF: Proximal Humerus Fracture(s)
PPI: Proton Pump Inhibitor
OR: Operating Room (if used in your text; adjust if needed)

## CRediT authorship contribution statement

**Lorenzo Lucchetta:** Conceptualization, Data curation, Investigation, Methodology, Writing – original draft. **David Palmieri:** Data curation, Investigation. **Michele Bisaccia:** Conceptualization, Supervision, Validation, Writing – review & editing. **Giuseppe Rinonapoli:** Conceptualization, Supervision, Validation, Writing – review & editing.

## Consent for publication

Written informed consent for publication of the case details and images was obtained from the patient.

## Ethics approval and consent to participate

Not applicable. This study did not require ethics approval because it reports a single case managed according to standard clinical practice and includes no experimental procedures.

## Funding

No funding was received for this study.

## Declaration of competing interest

The authors declare that they have no competing interests.

## Data Availability

All data generated or analysed during this study are included in this published article.
